# The Maternal and Child Health Handbook for Improving the Continuum of Care and Other Maternal and Child Health Indicators in Angola: An Implementation Study Protocol

**DOI:** 10.3389/fgwh.2021.638766

**Published:** 2021-03-25

**Authors:** Ai Aoki, Keiji Mochida, Michiru Kuramata, Toru Sadamori, Helga Reis Freitas, João Domingos da Cunha, Pedro Sapalalo, Lino Tchicondingosse, Olukunmi Omobolanle Balogun, Hisakazu Hiraoka, Hirotsugu Aiga, Kenji Takehara

**Affiliations:** ^1^Department of Health Policy, National Center for Child Health and Development, Tokyo, Japan; ^2^TA Networking Corp., Tokyo, Japan; ^3^Department of Global Health, Graduate School of Health Sciences, University of the Ryukyus, Nishihara, Japan; ^4^Samauma Consulting LLC, Tokyo, Japan; ^5^National Directorate of Public Health, Ministry of Health, Luanda, Angola; ^6^Domus Custodius (SU) Lda. Tchikos Agency, Luanda, Angola; ^7^Japan International Cooperation Agency, Tokyo, Japan; ^8^School of Tropical Medicine and Global Health, Nagasaki University, Nagasaki, Japan

**Keywords:** maternal and child health, maternal and child health handbook, home-based record, developing country, Angola, implementation

## Abstract

**Background:** Reducing maternal, neonatal, and infant mortality tops the health targets of sustainable development goals. Many lifesaving interventions are being introduced in antenatal, delivery, and postnatal care. However, many low- and middle-income countries (LMICs) have not reached maternal and child health targets. The Maternal and Child Health Handbook (MCH-HB) is recommended as a home-based record to promote a continuum of care from pregnancy to early childhood, and is gaining increasing attention among LMICs. Several countries have adopted it as national health policy. To effectively utilize the MCH-HB in LMICs, implementation needs to be considered. Angola is an LIMC in Sub-Saharan Africa, where maternal and child health indicators are among the poorest. The Angolan Ministry of Health adopted the MCH-HB program in its national health policy and is currently conducting a cluster randomized controlled trial (MCH-HB RCT) to evaluate its impact on the continuum of care. This study aimed to evaluate implementation status, and barriers and facilitators of MCH-HB program implementation in Angola.

**Methods:** To evaluate implementation status comprehensively, the RE-AIM (reach, effectiveness, adoption, implementation, and maintenance) framework will be used. Four components other than effectiveness will be investigated. A cross-sectional survey will be conducted targeting all health facilities and officers in charge of the MCH-HB at the municipality health office in the intervention group after the MCH-HB RCT. Data from the cross-sectional survey, secondary MCH-HB RCT data, and operational MCH-HB RCT records will be analyzed. Health facilities will be classified into good-implementation and poor-implementation groups using RE-AIM indicators. To identify barriers to and facilitators of MCH-HB implementation, semi-structured interviews/focus group discussions will be conducted among health workers at a sub-sample of health facilities and all municipality health officers in charge of MCH-HB in the intervention group. The Consolidated Framework for Implementation Research will be adopted to develop interview items. Thematic analysis will be performed. By comparing good-implementation and poor-implementation health facilities, factors that differ between groups that contribute to successful implementation can be identified.

**Discussion:** This study's findings are expected to inform MCH-HB implementation policy and guidelines in Angola and in other countries that plan to adopt the MCH-HB program.

## Introduction

Maternal and child health, especially maternal, neonatal, and infant mortality, is among the highest public health priorities in many low- and middle-income countries (LMICs). The United Nations's Sustainable Development Goals (SDGs) list maternal and child health at the top of SDG target 3: Ensure healthy lives and promote well-being for all at all ages (1). However, many LMICs have not yet achieved their targets, especially those in Sub-Saharan Africa (2, 3).

To improve maternal and child health, promotion of a continuum of care (CoC) from pregnancy and delivery to early childhood is essential in addition to provision of essential lifesaving services (4, 5). Education of mothers, families, and communities is a key intervention for promotion of CoC (6).

The Maternal and Child Health Handbook (MCH-HB) is an integrated home-based record (HBR) and is designed to record all the key information and data of health service utilization and health conditions of a mother and her child during the course of pregnancy, delivery, and after birth (e.g., maternal care and the child's growth and immunizations) (7, 8). In addition to the aforementioned uses, the MCH-HB functions as a self-learning resource, helps avoid multiple HBRs (9), and supports improvements in CoC (10, 11). As a result, the MCH-HB has been drawing greater attention from health ministries and professional organizations as an effective tool for promoting a life-course approach to health care (8). The MCH-HB has been introduced in more than 50 countries (e.g., Indonesia, Mongolia, the Philippines, and Sudan) (7, 12).

The MCH-HB program was adopted in Angola in a national health policy to increase CoC, with technical support from the Japan International Cooperation Agency (“Project for Improving Maternal and Child Health Services through implementation of the Maternal and Child Health Handbook”). The MCH-HB program is a package of MCH-HB distribution and health worker education to utilize it effectively. Preceding its nationwide scaling-up, a cluster randomized controlled trial (MCH-HB RCT) aimed at estimating the impacts of the MCH-HB program on CoC achievements has been conducted in one province, starting in June 2019 (13).

Better implementation of an evidence-based intervention is key for health promotion in LMICs (14). However, evidence related to the implementation of the MCH-HB program, which refers to implementation of the entire package, is still lacking (15). To better implement the MCH-HB program and to better understand its impact on health systema and health workers, this study aimed to evaluate the implementation status of the MCH-HB program and its barriers and facilitators in the intervention group of the MCH-HB RCT. Identification of barriers to and facilitators of the MCH-HB program will provide other provincial health departments in Angola with useful insights on more effective and efficient implementations of the MCH-HB program. As some other countries are considering including the MCH-HB in their national policies, barriers to and facilitators of implementation represent essential information for countries that plan to adopt the MCH-HB program.

## Methods and Analysis

### Study Setting

This study will be conducted in Angola's Benguela province. Angola is a lower middle-income country in Sub-Saharan Africa (16). According to World Health Organization (WHO) estimates, approximately 477 maternal deaths occurred per 100,000 live births in 2015 (2), primarily due to preventable diseases and other health problems. In addition, Angola remains one of the African countries with the highest burden of under-5 mortality [81 per 1,000 live births] and infant mortality rates [54 per 1,000 live births], despite a consistent reduction during recent years (17). Some contributors to the inadequate achievements in maternal and child health indicators in Angola include lower functioning health systems and shortfalls in the health workforce (18, 19).

Benguela is located in the southwest of the country, facing the Atlantic Ocean. Benguela has 10 administrative divisions called municipalities (20), and a population of ~2.2 million (21), being the third most populous province in Angola. Benguela was purposively selected as a site for the MCH-HB RCT because data on major health indicators are at a similar level to the national average. Benguela is representative enough to show the impact of the MCH-HB program. The MCH-HB program is taking place in all health facilities that provide maternal, neonatal, and child health services (MNCH services) in the intervention group of the MCH-HB RCT. Health facilities are categorized into three levels: primary, secondary, and tertiary. The MCH-HB RCT started in June 2019, and an endline survey took place until October 2020.

### Intervention

**Figure 1:**
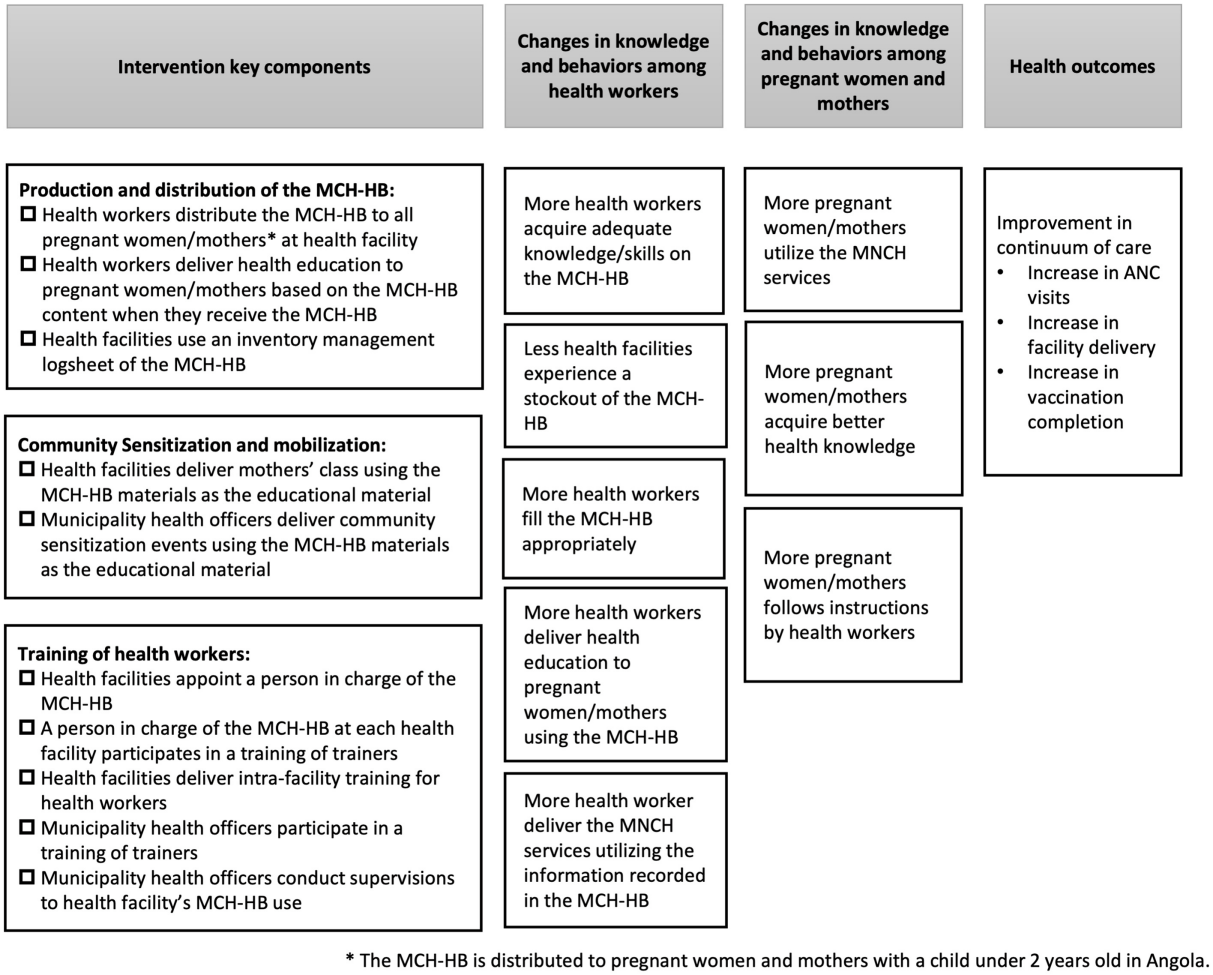
Logic model for the MCH-HB to improve continuum of care among pregnant women and mothers.

The intervention has three components: distribution of the MCH-HB to pregnant women at health facilities designated as the MCH-HB distribution points in the intervention arm, training of health workers on MCH-HB operation, and community sensitization and mobilization targeting pregnant women on the use of the MCH-HB (Figure 1). Regarding the first component, the MCH-HB is provided to all pregnant women and mothers at the time of their first visits to health facilities for the purpose of receiving MNCH services, and the recipients receive health education based on MCH-HB content and an instruction to bring the handbook to every visit. To ensure distribution of the handbook to all eligible women, inventory management of the MCH-HB to avoid stockout is also instructed. Regarding the second component, representatives of each health facility completed the training of trainers prior to the beginning of the MCH-HB RCT. Those who completed the training of trainers were responsible for further undertaking internal training targeting health workers at their duty station health facilities. Municipality officers from each municipality health office who were responsible for the MCH-HB program participated in the training of trainers prior to the MCH-HB RCT and were supposed to conduct an inspection of the management of the program at each health facility in their municipality. Regarding the third component, each health facility was advised to provide mothers' classes using MCH-HB material as the education material, while municipality officers were instructed to hold community mobilization events using MCH-HB material. CoC was assessed as an effectiveness outcome indicator at MCH-HB RCT. The details of the intervention and of usual care are described elsewhere (13).

### Implementation Study Design

This implementation study will be carried out in five municipality health offices and all 99 health facilities in the five municipalities allocated to the intervention arm. The data will be collected via a combination of cross-sectional surveys and secondary data sources. To assess the implementation status of the MCH-HB program, a cross-sectional survey will be conducted targeting all 99 health facilities and five municipality health offices. A quantitative survey to evaluate health workers' skills and knowledge will be conducted among all health facilities. To identify the barriers to and facilitators of implementation of the MCH-HB program, semi-structured interviews/focus group discussions will be conducted among health workers at 25 selected health facilities and all municipality health officers responsible for the program. The data from the MCH-HB RCT and operational records at the health facility will be used as secondary data sources.

### Data Collection

#### Evaluation of MCH-HB Implementation Status

A questionnaire will be completed by health workers in charge of the MCH-HB at each health facility and municipality health officers in charge of the program at each municipality health office. An originally developed exam will be used to evaluate the skills and knowledge regarding the MCH-HB program among health workers (health workers in charge of MCH-HB as well as health workers in non-management positions). The target number of participants is set at one per health facility for health workers in charge of the program and three per health facility for those in non-management positions. Depending on number of staff and the facility's work conditions, a feasible number of health workers will be recruited, aiming at the target number. An exam previously used for the training of trainers in the preparatory phase of the MCH-HB RCT will be employed. All examinees were required to achieve 70/100 points during the training of trainers. A quantitative survey will be conducted to evaluate the subjective burden of the MCH-HB program among health workers. The target number of participants is the same as for the exam.

#### Identification of the Barriers and Facilitators of MCH-HB Implementation

Semi-structured interviews/focus group discussions will be conducted to identify the barriers and facilitators of implementation of the MCH-HB program. At the municipality health office, an individual interview will be conducted with municipality health officers in charge of the program. At health facilities, an individual interview will be conducted with the manager, and a focus group discussion will be conducted among department directors and health workers in non-management positions (Table 1).

**Table 1:** Interview/focus group discussion participants and the total number of interviews/focus group discussions.

	**Type of participants (Target number of participants)**						**Total number of interviews/** **discussions per facility**	**Number of facilities**	**Total number of interviews/** **discussions**
	**Manager**	**Antenatal care department director**	**Delivery department director**	**Postnatal care/vaccination department director**	**Health workers in non-management positions**	**Municipal health officer**			
Primary health facility	Individual interview				Focus group discussion		2	10	20
(two facilities/municipality)	(one participant)				(four participants)				
Secondary health facility	Individual interview	Focus group discussion			Focus group discussion		3	10	30
(two facilities/municipality)	(one participant)	(three participants)			(four participants)				
Tertiary health facility	Individual interview	Focus group discussion			Focus group discussion		3	5	15
(one facility/municipality)	(one participant)	(three participants)			(four participants)				
Municipality						Individual interview			5
						(one participant)			

### Frameworks and Data Analysis

#### Evaluation of MCH-HB Implementation Status

The RE-AIM framework will be used to assess the implementation status of the MCH-HB program (22). RE-AIM is a framework to evaluate implementation through five constructs: (1) reach, (2) effectiveness, (3) adoption, (4) implementation, and (5) maintenance. As (2) effectiveness will be evaluated in the MCH-HB RCT, the other four constructs will be evaluated in this study. Among the three components of the MCH-HB program, distribution is evaluated in (1) the reach construct, while training of health workers and community sensitization and mobilization are mainly evaluated in (3) the adoption and (4) implementation constructs. For continuous health facility indicators, a target level was set according to a previous survey on HBRs and expert opinion (23) (Table 2).

**Table 2:** Implementation variables and constructs.

**Construct**	**Indicator**	**Unit**	**Definition**	**Target**	**Data source**
Reach	MCH-HB coverage	Health facility	% MCH-HB distribution among new visitors to antenatal care services	95%	Health facility survey
		Municipality	% MCH-HB receivers among the estimated number of pregnant women during the study period in the municipality		Baseline survey^*^, census
Adoption	Training	Health facility	Participation in the training of trainers	Yes	Project operational records
		Health facility	Holding an intra-facility training	Yes	Health facility survey
	Inventory management	Health facility	Use of inventory management sheets	Yes	Health facility survey
	Mothers' class	Health facility	Holding mothers' classes every week	Yes	Health facility survey
	Community sensitization/ mobilization	Municipality	Holding community sensitization/mobilization activities using MCH-HB material		Municipality survey
			Number of community sensitization/mobilization activities		Municipality survey
	Supervision	Municipality	% health facilities that received supervision by a municipal health officer		Health facility survey
Implementation	MCH-HB retention	Health facility	% MCH-HB holders at the end of the trial among MCH-HB receivers	90%	Baseline survey, endline survey^*^
	MCH-HB utilization	Health facility	% appropriate birth weight description among MCH-HB receivers	80%	Endline survey^*^
	Inventory management	Health facility	Stockout	No	Health facility survey
	Mothers' class	Health facility	Holding mothers' class according to the instructions on themes	Yes	Health facility survey
Maintenance	Intra-facility training	Health facility	Definite person in charge of intra-facility training after MCH-HB RCT	Yes	Health facility survey
	Skills and knowledge	Health facility	Median score of health workers in charge of MCH-HB above the required level	70/100	Health facility survey
		Health facility	Median score of health workers in non-management positions above the required level	60/100	Health facility survey
	Subjective burden	Health facility	Subjective burden of at least one health worker in charge of MCH-HB being “low” or “very low”	Yes	Health facility survey
		Health facility	Subjective burden of at least one health worker in non-management positions being “low” or “very low”	Yes	Health facility survey
	Introduction to new health facility	Municipality	% new health facilities that introduced MCH-HB among all the new health facilities		Project operational records

* *Baseline and endline survey is baseline and endline survey of MCH-HB RCT*.

“Reach” refers to the extent to which an intervention reaches its target population. Reach is important, as the MCH-HB should be distributed to all pregnant women. Coverage of the MCH-HB at health facilities in September 2020 as well as among all pregnant women in the municipality/region during the study period will be assessed. Coverage of the MCH-HB at health facilities will be assessed among new antenatal care service users. Coverage of the MCH-HB among antenatal care service users is defined as the proportion of the number of MCH-HB distributed at antenatal care services to the number of new visits to antenatal care service during September 2020; this data will be extracted from health facility records. Coverage of the MCH-HB among all new MNCH service users cannot be assessed because health facility records do not distinguish the first visit to delivery/postnatal care services and the first visit to entire MNCH services. For example, women who visit a child health service for the first time may have visited antenatal care services previously, but would still be recorded as a first visitor to the child health service. The target level for coverage at the health facility level is set at 95%, which is required to achieve a desirable community coverage of HBR (90%) at the municipality level under the condition that the health facility utilization rate for receiving antenatal care services still has room to improve (23). Coverage of the MCH-HB among all pregnant women in the municipality during the study period is defined as the proportion of the total number of MCH-HB distributed at MNCH services to the estimated number of pregnant women in the municipality/region during the study period. The total distribution number will be obtained from the MCH-HB RCT data. To estimate the number of pregnant women in the municipality/region, the census data from 2015 will be used (21). The census in 2015 constitutes the latest municipality level data available on the number of pregnant women.

“Adoption” refers to adoption of components of the MCH-HB program at the health facility and municipality health office levels. At the health facility level, participation in the training of trainers, intra-facility training, utilization of inventory management sheets, and provision of mothers' classes will be assessed. These are all binary indicators. At the municipality health office level, the provision of community sensitization/mobilization events and supervision of health facilities will be assessed.

As an “implementation” construct, implementation fidelity at the health facility level will be assessed. The fidelity of training of health workers will be evaluated by retention of the MCH-HB among MCH-HB RCT participants (MCH-HB retention) as well as by an appropriate description of the child's birth weight among MCH-HB RCT participants (MCH-HB utilization) (23). The target level for MCH-HB retention is set at 90% and MCH-HB utilization is set at 80% according to the expert opinion. Fidelity of inventory management will be assessed by stockout of the MCH-HB during the MCH-HB RCT. Fidelity of mothers' classes will be assessed by the theme of the mothers' classes. The health facility is instructed to provide mothers' classes with multiple themes using MCH-HB program material. Indicators for inventory management and mothers' classes are binary indicators.

“Maintenance” refers to factors that influence sustainability of the MCH-HB program at a health facility. At the health facility, the training system after the MCH-HB RCT, skills and knowledge necessary for appropriate operation of the MCH-HB program, and the subjective burden of MCH-HB program use will be assessed. To evaluate skills and knowledge, the same exam that was used to evaluate trainer training will be used. The same target score is set for health workers in charge of the MCH-HB program (70/100 points), and a lower score is set for health workers in non-management positions (60/100 points). The subjective burden will be evaluated on a 5-point Likert scale. Introduction of the MCH-HB program at new health facilities established during the MCH-HB RCT period will also be assessed as an indicator of maintenance at municipality level.

#### Identification of the Barriers and Facilitators of MCH-HB Implementation

The Consolidated Framework for Implementation Research (CFIR) will be adopted as a framework for the analysis (24, 25). CFIR has five domains: (1) intervention characteristics, (2) outer setting, (3) inner setting, (4) characteristics of individuals, and (5) process. The intervention characteristics domain refer to key attributes of interventions that influence the success of implementation (25). The outer setting domain refers to outer setting including the economic, political and social context within which an organization resides (24). The inner setting domain refers to features of structural, political, and cultural contexts of the organization where the intervention is implemented (24). The characteristics of individuals domain concerns individuals who are involved with the intervention and/or implementation process (24). The process domain refers to a change process that aims to achieve individual and organizational level use of the intervention as designed (24). Facilitators used a few main questions for each CFIR domain and topics list which were developed based on constructs. Key questions are such as the difference between MCH-HB and conventional tools for the intervention characteristics domain, external factors influencing the implementation for the outer setting domain, organizational features influencing the implementation for the inner setting domain, health workers' ability to utilize MCH-HB as it is designed for the characteristics of individual domain, and the feasibility of the plan and problems in execution for the process domain. All semi-structured interviews and focus group discussions will be facilitated by a group of research assistants. All semi-structured interviews and focus group discussions will be conducted in the local language, Portuguese. Their contents will be audio-recorded and transcribed. Transcriptions will be translated into English.

### Data Analysis

#### Evaluation of MCH-HB Implementation Status

Health facility level implementation variables will be descriptively analyzed together as well as by sub-categories of health facilities such as municipality and the health facility level. Municipality level implementation variables will be analyzed together as well as by municipality.

At the health facility level, global implementation status is evaluated using health facility implementation variables. Continuous variables will be converted into binary variables using the target level as a threshold, and the total score of implementation status will be calculated. The total score ranges from zero to 14. The target score is set at nine out of 14 based on the expert opinion. In case some health facility implementation variables are missing, to assess global implementation status the achievement rate will be calculated, removing missing values from both numerator and denominator. The target achievement rate is set at 65%. If the number of missing values among health facility implementation variables is larger than seven, global implementation status will not be assessed. According to the target score and target achievement rate, health facilities will be categorized into good-implementation and poor-implementation groups. If the target score and target achievement rate categorize health facilities in an imbalanced way, and the proportion of smaller group becomes <10%, the median score of achievement rate among all health facilities will be used to categorize health facilities.

#### Identification of the Barriers and Facilitators of MCH-HB Implementation

Translated transcriptions from interviews/focus groups will be coded according to the CFIR framework. A researcher will code them, and the codes and categories which will be extracted will be confirmed by several experts who well-understand the situation in Angola and MCH-HB program in other countries. Health facilities and municipalities will be analyzed separately. Health facilities will be categorized into good-implementation and poor-implementation health facility groups. Good-implementation and poor-implementation health facilities will be compared and key barriers/facilitators will be identified. Key barriers and facilitators for program implementation at the municipality and at the health facility levels will be identified.

## Discussion (Practical Operational Issues)

This paper is a protocol for an implementation study to assess the implementation status and its barriers and facilitators of the MCH-HB program in Angola. This study will demonstrate the implementation performance of the MCH-HB program and its barriers and facilitators. The evidence generated through this research will be utilized to better scale-up the MCH-HB program in Angola and to better implement the MCH-HB program in other countries.

## Limitation

This study approaches health facilities, health workers and municipality health officers and does not approach users. Even though the user perception will be asked in the interviews/focus group discussions among health workers and municipality health officers, barriers and facilitators from the user perspective will not be enough captured. This will be a future research focus.

## Ethics Statement

The studies involving human participants were reviewed and approved by National Center for Child Health and Development, Japan the Ministry of Health of the Republic of Angola, Angola. The patients/participants provided their written informed consent to participate in this study.

## Author Contributions

AA, KT, KM, MK, OB, HA, and HH developed the concept and design of this study. AA and KT drafted the first manuscript. PS, LT, HF, JC, OB, KM, MK, and TS coordinated the local logistics, including training of research assistants and management of data. All authors read, contributed to, and provided a critical review of the final manuscript.

## Conflict of Interest

KM was employed by the company TA Networking Corp. MK and TS were employed by the company Samauma Consulting LLC. PS and LT were employed by the company Domus Custodius (SU) Lda. Tchikos Agency. The remaining authors declare that the research was conducted in the absence of any commercial or financial relationships that could be construed as a potential conflict of interest.
